# The Impact of Yoga Practices on Body Composition and Vital Signs in Children and Adolescents With Obesity: A Systematic Review and Meta‐Analysis

**DOI:** 10.1111/obr.13947

**Published:** 2025-05-19

**Authors:** Nuray Caner, Gülyeter Erdoğan Yüce

**Affiliations:** ^1^ Faculty of Health Sciences Erciyes University Kayseri Türkiye; ^2^ Faculty of Health Sciences Aksaray University Aksaray Türkiye

**Keywords:** adolescents, children, meta‐analysis, obesity management, yoga

## Abstract

**Introduction:**

Clear recommendations for treating childhood obesity include family involvement, improving diet quality, reducing portion sizes, increasing physical activity, and decreasing sedentary behaviors.

**Methods:**

This systematic review and meta‐analysis aimed to provide evidence the effects of yoga interventions on BMI, weight, body composition, and vital signs in children or adolescents with overweight/obesity. This study searched the PubMed, Web of Science, and EBSCO databases to include all studies published up to June 2024. Methodological quality was assessed with the RoB 2. Randomized or nonrandomized controlled studies were included. The standardized mean difference with a 95% confidence interval was calculated. Heterogeneity was analyzed using the *I* ^2^ test and *Q* statistic. Publication bias was assessed with the Egger regression test.

**Results:**

This meta‐analysis included nine studies. Pooled evidence found that yoga significantly reduces BMI and body fat percentage in children or young people with overweight/obesity.

**Conclusion:**

In conclusion, findings derived from pooled data yoga intervention is an effective method for weight management in children and adolescents with obesity. Additionally, considering that yoga is a calming and mind–body‐based intervention, it is recommended that future studies also examine the psychological effects of yoga in children and adolescents with obesity.

## 1 Introduction

Obesity, resulting from the interplay of physiological, psychological, environmental, socioeconomic, and genetic factors, is a significant global health issue [1]. In children and adolescents, overweight and obesity are among the risk factors for health problems and cardiovascular diseases, and they represent a significant health issue contributing to the global rise of noncommunicable diseases [2]. In recent decades, there has been a substantial increase in obesity prevalence, with one in every eight individuals categorized as obese as of 2022 [1]. The World Obesity Federation estimates that by 2025, 206 million children and adolescents aged 5 to 19 will be living with obesity, with this figure projected to rise to 254 million by 2030 [3].

The risk of developing various comorbidities increases due to obesity. The presence of obesity during childhood and adolescence has been reported to contribute to the development of type 2 diabetes and hypertension and significantly impact the decline in overall quality of life. Moreover, it has been shown that physical and mental health outcomes related to obesity may persist into adulthood [4]. When examining the short‐ and long‐term effects of obesity on the body, it is found to increase the risk of developing various physical and mental health conditions, particularly cardiovascular diseases. Obesity is a disease resulting from the interplay of genetic, environmental, psychological, socioeconomic, and hormonal factors, characterized by excessive fat accumulation in metabolic tissues and organs. Also, obesity can significantly decrease the quality of life and life expectancy by releasing various cytokines and adipokines. Obesity negatively affects children's psychosocial development, leading to reduced self‐esteem, stigmatization, and deteriorating mental health, which can persist into adulthood [2, 5]. Martin et al. [6] have highlighted the relationship between excess body fat percentage and cognitive development in children, noting that childhood obesity affects attention and cognitive functions, leading to poor academic performance and socioeconomic challenges.

Weight management strategies for children with obesity are based on behavior modification, physical activity and nutrition [7]. Clear recommendations for treating obesity include family involvement, improving diet quality, reducing portion sizes, increasing physical activity, and decreasing sedentary behaviors [8, 9]. In this context, to effectively manage all these interventions and achieve successful outcomes, a multidisciplinary team consisting of physicians, physiotherapists, nutritionists, and nurses could give guidance to reduce obesity risk factors [2]. Various treatment approaches exist for obesity and overweight, including educational programs, family involvement programs, school‐based programs, mindfulness‐based interventions, and physical exercise programs [6]. Implementing physical activity programs to reduce BMI can facilitate weight management in the challenging and prolonged treatment of obesity. Various approaches based on physical activity are found in the literature. Yoga therapy is also one of these physical activity interventions [10].

Yoga therapy offers holistic benefits for physical, mental, and emotional health [11]. Traditionally, yoga promotes overall well‐being by harmonizing the body, mind, and spirit. The components of yoga include asana (stretching movements), pranayama (breathing exercises), and dhyana (meditation) [12]. Asana refers to physical movements specific to yoga, which also yield mental and cognitive effects. Asanas enhance the individual's emotional, physical, and mental well‐being, support increased energy. Pranayama involves consciously regulating the movement of the lungs to establish balance between inhalation and exhalation. This regulation creates positive effects on facilitating control over the autonomic nervous system and regulating physiological functions. Dhyana, or meditation, increases the level of awareness and helps to still the mind [12, 13]. It has been reported that yoga therapy improves physical strength and the functions of the cardiovascular and respiratory systems, increases metabolic rate and is effective in weight management while minimizing cardiovascular risk factors [14]. A systematic review and meta‐analyses on the effects of yoga on weight‐related outcomes exist in the literature [15, 16]. However, the study stated that the effects of yoga on obesity‐related outcomes cannot be interpreted for children and adolescents [16]. With the growing interest in yoga, there is a need for meta‐analytic studies to determine the effects of yoga on body composition and metabolic parameters in children and adolescents with obesity.

## 2 Method

### 2.1 Study Design

The reporting of this study was carried out according to the “Preferred Reporting Items for Systematic Reviews and Meta‐Analyses (PRISMA)” guidelines [17]. The research questions were formulated using the Population, Intervention, Comparison, and Outcome (PICO) strategy [18]. The participants (P) of the meta‐analysis consisted of individuals aged 10–24 with obesity. The intervention (I) included yoga practices, and the comparison (C) involved other exercise programs, no intervention, or standard treatment methods. The outcome variables included body composition (BMI, weight, body fat) and vital signs (blood pressure, heart rate). The research question formulated according to the PICO strategy is: “Is yoga applied to children and young people with obesity effective on body composition and vital signs compared to other exercise programs or no intervention?”

### 2.2 Data Sources and Search Strategy

This study searched the PubMed, Web of Science, and EBSCO databases to include all studies published up to June 2024 without any year restrictions. The literature search was conducted using the keywords “Yoga” AND “BMI” OR “Body Mass Index” OR “Obese” OR “Weight” OR “Obesity” OR “Overweight” AND “Child” OR “Youth” OR “Adolescent” OR “Pediatric” OR “Youth”.

### 2.3 Data Extraction

The sources from the databases were imported into Mendeley and systematically reviewed by the researchers. After removing duplicate studies, the researchers independently evaluated the remaining studies' titles and abstracts based on the inclusion and exclusion criteria. The characteristics and results of the included studies were summarized in a standard form. This form included the author(s), publication year, country, sample, sample size, participants' age, participants' sex (%), intervention duration, intervention strategy, control strategy, assessment time, and outcomes. If there were inconsistencies in the selected studies and the extracted data, the researchers discussed the issue and collaborated to resolve it.

### 2.4 Inclusion and Exclusion Criteria

Studies published in English, with randomized or nonrandomized controlled design, including obese individuals aged 10–24 years, were included in this meta‐analysis. No restriction was made on sample size. The age range was classified according to WHO. According to this classification, obese individuals aged 10–24 were included in this meta‐analysis. Individuals are considered young and are evaluated within the scope of adolescent health [19]. Studies published in languages other than English, those that did not report mean (M) and standard deviation (SD) values, secondary analyses, systematic reviews and meta‐analyses, theses, conference proceedings, short reports, and studies in the gray literature (e.g., preprints and technical reports) were excluded. Only peer‐reviewed articles published in scientific journals were included.

### 2.5 Quality Assessment

The quality of the studies was assessed independently by the researchers using the Revised Cochrane Risk of Bias Tool (RoB 2), developed by the Cochrane Collaboration. The researchers discussed and resolved any inconsistencies in the assessments. The RoB 2 tool assessed bias due to the randomization process, deviation from the intended interventions, and missing results. It consists of five domains: bias in data, bias in the measurement of outcome, and bias in the selection of reported outcome. Each domain is categorized as “high risk of bias,” “some concern,” or “low risk of bias” [20].

### 2.6 Data Synthesis

Data were analyzed using the MAJOR package in JAMOVI 2.3.28 [21]. As the measurements in the studies included in the meta‐analysis were performed using standard units of measurement, the effect size was measured using the mean difference (MD) with a 95% confidence interval (CI). Forest plots were created to represent the MD with 95% CI visually, and the overall effect size was calculated. The z‐value represented statistical significance. Heterogeneity among studies was assessed using the *Q* statistic and *I* ^2^ statistics. The *Q* statistic tests the null hypothesis of homogeneity, assuming a chi‐square distribution with (k—1) degrees of freedom for k studies. A significant *Q* statistic (*p* < 0.05) indicates the presence of heterogeneity among the studies. *I* ^2^ values above 50% indicate significant heterogeneity, while values below 50% indicate low or insignificant heterogeneity. When the *Q* statistic and *I* ^2^ values did not indicate heterogeneity, a fixed‐effect model was used in the analysis; when heterogeneity was indicated, a random‐effects model was used [22]. Egger's regression, funnel plots, and the Begg and Mazumdar rank correlation tests assessed publication bias [23, 24]. A significance level of *p* < 0.05 was considered for all statistical evaluations.

## 3 Results

### 3.1 Search Outcomes

**FIGURE 1:**
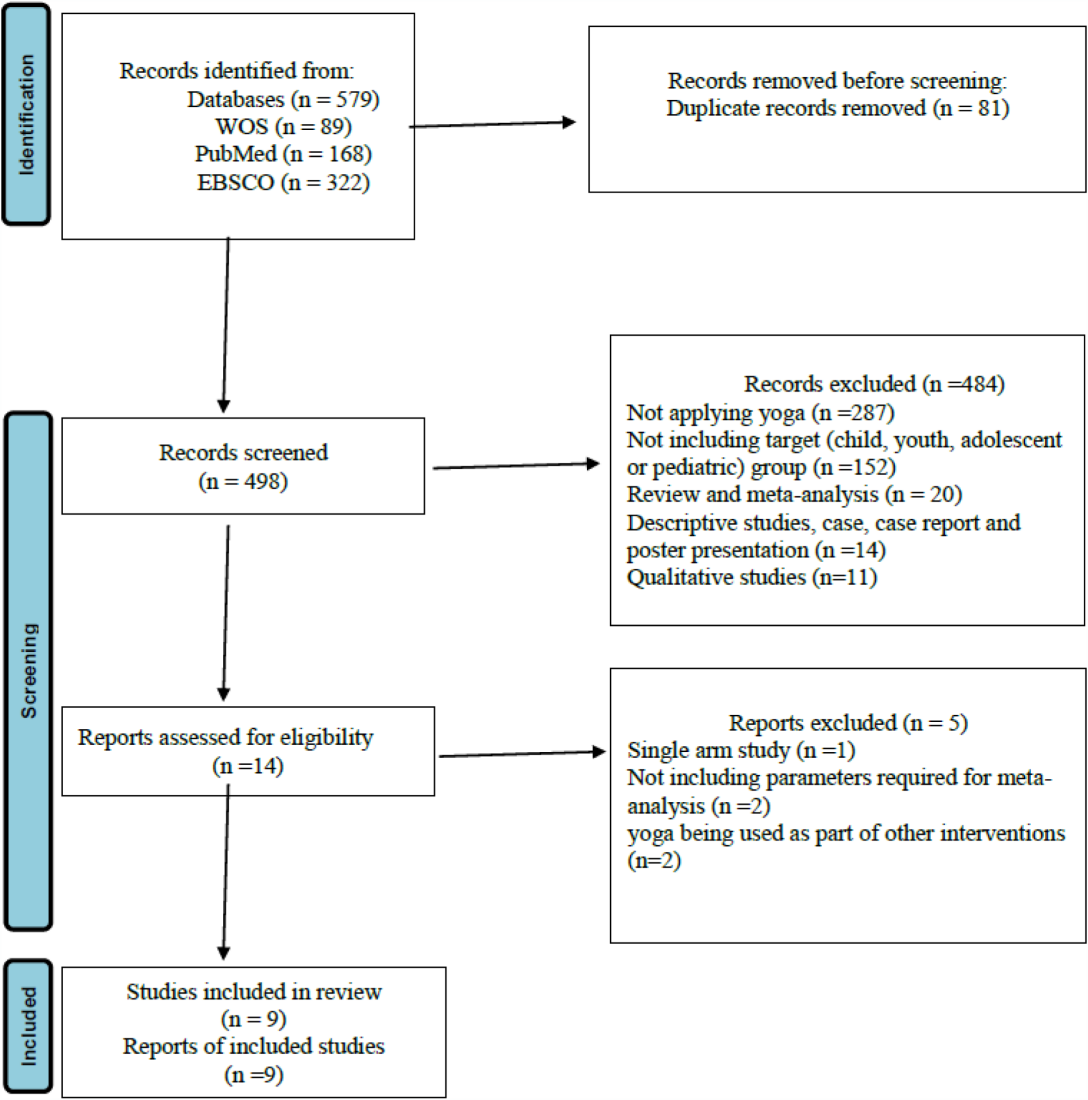
PRISMA flow diagram.

The PRISMA flow diagram summarized the literature search and exclusion criteria (Figure 1). A total of 579 references were retrieved from three databases. Eighty‐one duplicates were removed. The titles and abstracts of the remaining 498 studies were screened, excluding 484 studies. Fourteen studies remained. The full texts of these studies were assessed for inclusion criteria. A total of five studies were excluded for being single‐arm (*n* = 1), lacking parameters necessary for meta‐analysis (*n* = 2), and yoga being used as part of other interventions (*n* = 2). As a result, nine eligible studies were included in this meta‐analysis.

### 3.2 Characteristics of the Included Studies

The characteristics of the included studies and information related to the interventions are given in Table 1. The studies were conducted between 2012 and 2023. The nine included studies were conducted in the following countries: India (*n* = 5), China (*n* = 1), Thailand (*n* = 1), United States (*n* = 1), and South Korea (*n* = 1). The total number of participants was 319, with sample sizes ranging from 5 to 41. The participants' average age ranged from 10 to 22, and the duration of the interventions ranged from 1 month to 18 weeks.

**TABLE 1:** Characteristics of reviewed studies.

First author, year/country	Type of participants	Sample	Age (mean ± SD)	Sex (%)	Duration of intervention	Intervention strategies	Control strategies	Assessment times	Outcomes
[25]/India	Obese children	Yoga = 5 Control = 5	10.22 ± 0.78	NI	6 weeks	The intervention group underwent a structured Hatha Yoga training program of 40 min 3 days a week for 6 weeks.	No intervention	Baseline and post‐intervention	BMI HRQoL
[26]/China	Overweight or obese students in the first grade of a high school	Yoga = 20 Control = 20	Yoga: 16.01 ± 0.52 Control: 16.36 ± 0.34	50% Female	8 weeks	The experimental group did professional yoga training for 8 weeks according to the normal frequency of the school physical education class.	The control group participated in regular light sports activities in physical education class.	Baseline and Post‐intervention	BMI Weight Body fat Area of visceral fat Quiet heart rate Quiet blood pressure Pulmonary function Mental health
[27]/India	Obese female adolescents	Yoga = 20 Diet = 20 Control = 20	Yoga: 19.7 ± 5.3 Diet: 19.9 ± 4.3 Control: 20.2 ± 2.6	100% Female	12 weeks	The yoga group received three 60‐min sessions per week that included postures, breathing exercises and relaxation techniques.	Participants continued their daily activities without any intervention.	Baseline and Post‐intervention	Weight BMI Body fat mass Body fat percentage
[28]/USA	Pediatric population with obesity	Yoga = 6 Control = 10	Yoga: 11.8 ± 1.6 Control: 11.2 ± 1.8	50% Female	12 weeks	The yoga group received 60 min of didactic training and 45–60 min of hatha yoga sessions once a week.	The active control group received 60 min of didactic training and 45–60 min of physical activity once a week.	Baseline and Post‐intervention	Weight Height BMI Health habits
[29]/India	Children and adolescents with obesity	Yoga = 41 Weight management = 40 Control = 28	11.6 ± 1.8	32.2% Female	18 weeks	There was an intensive phase where the yoga group had to attend at least three but preferably five classes a week for 2 weeks. It was necessary to attend a class once a week from the 3rd week to the 6th week and every 4 weeks from the seventh week to the 18th week. Starting from the third week, they were asked to do yoga at home for an hour at least five times a week. Detailed nutritional counseling was also provided.	Standard weight management (dietary and lifestyle counseling) and no intervention (control group).	Baseline, Post‐intervention and follow‐up at 6–12 months	BMI Weight Waist circumference Heart rate Blood pressure Dietary intake Stress BSQ Kindl QoL
[30]/Thailand	Obese adolescents	Yoga = 20 Control = 20	19–22 years	100% Female	12 weeks	The yoga group participated in a yoga program for 50 min three times a week for 12 weeks.	No intervention	Baseline, week 8 and Post‐intervention	BMI Body fat mass Muscle mass
[31]/India	Overweight obese adolescents	Yoga = 14 Control = 9	Yoga: 14.21 ± 1.84 Control: 15.22 ± 1.09	34.7% Female	One month	The yoga group participated in a yoga program for 60 min 5 days a week for 1 month.	The control group was doing regular physical activities. Other than that, there was no intervention.	Baseline, Post‐intervention	Weight BMI Heart rate Blood pressure Mid upper arm circumferences Abdominal circumference Waist circumference Hip circumference Fasting blood sugar HDL LDL VLDL Sr. cholesterol Triglycerides
[32]/India	Obese adolescents	Yoga = 30 Control = 30	Yoga: 11 ± 1.4 Control: 11 ± 1.3	55% Female	40 days	The yoga group participated in a yoga program for 60 min five times a week for 40 days.	No intervention	Baseline, Post‐intervention	Weight BMI Heart rate Blood pressure Mid upper arm circumferences Abdominal circumference Waist circumference Hip circumference
[33]/South Korea	Obese male adolescents	Yoga = 10 Control = 10	14.65 ± 0.74	100% Male	8 weeks	The yoga group participated in a yoga program for 50 min three times a week for 8 weeks.	No intervention	Baseline, Post‐intervention	Weight BMI Fat‐free mass Fat mass Body fat BMR TC TG HDLc LDLc HDLc/LDLc Glucose Insulin HOMA‐IR

### 3.3 Risk of Bias

**FIGURE 2:**
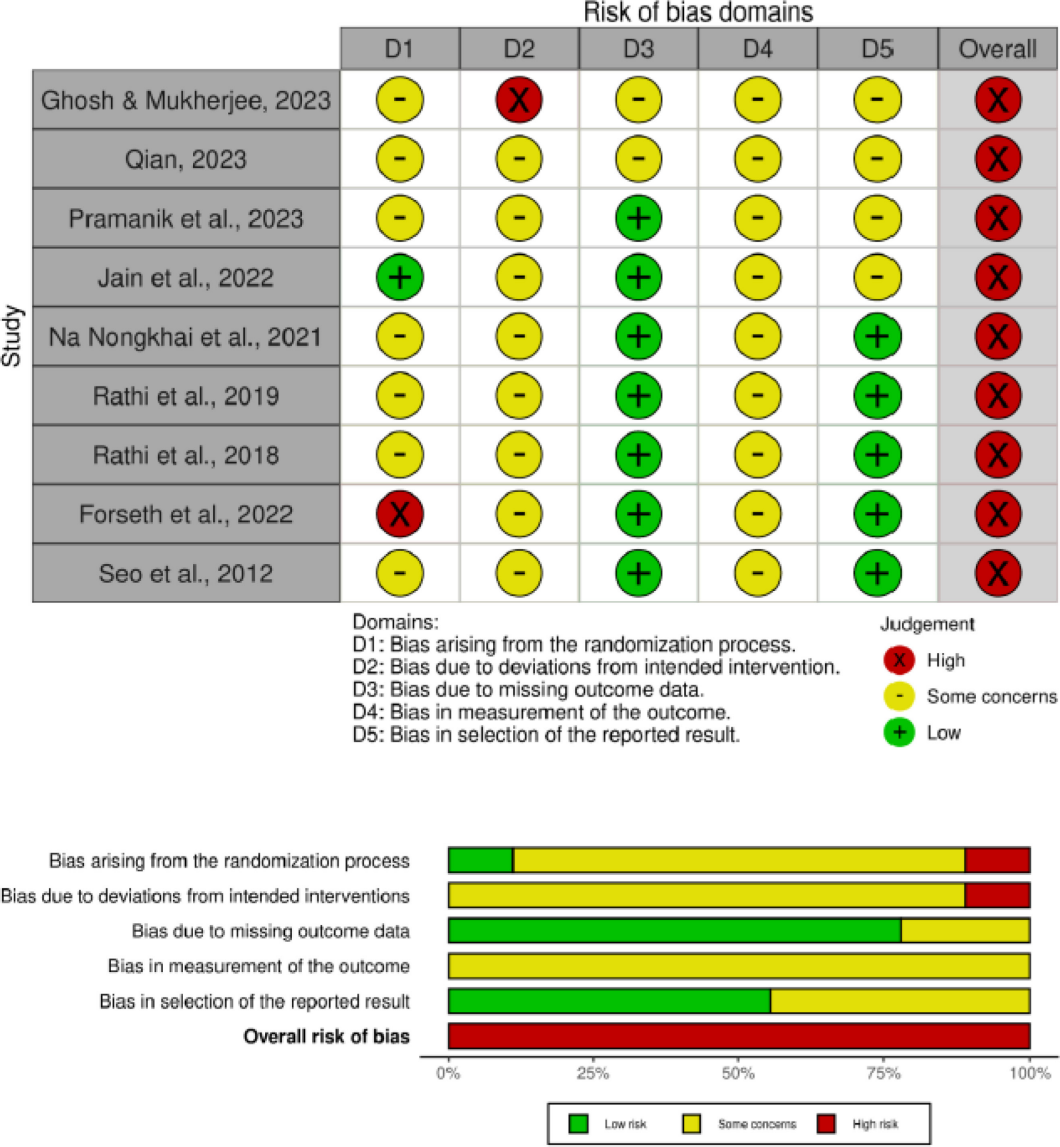
Risk of bias assessment for included studies.

The risk of bias in the included studies is shown in Figure 2. Randomization was reported in eight of the included studies, but details regarding randomization were provided in only one study (11.1%). Eight studies (88.8%) were concerned about the risk of bias due to deviations from intended interventions. Two studies (22.2%) were concerned about the risk of bias due to missing outcome data. All studies had concerns about the risk of bias in measuring the outcome. The risk of bias in selecting the reported result was low in five studies (55.5%). Overall, all studies were determined to have a high risk of bias.

### 3.4 Effects of Yoga on Outcomes

**FIGURE 3:**
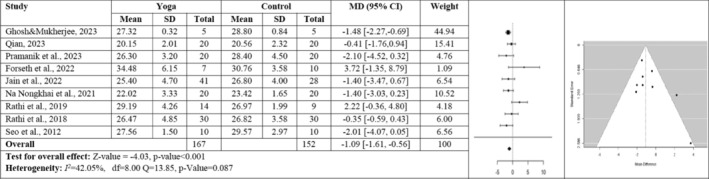
Forest plot and funnel plot for the BMI of the yoga and control group.

Nine studies, including 319 participants examining the effect of yoga on BMI, were included in the analysis. *Q* test and *I* ^2^ for heterogeneity was not significant (*Q*(8) = 13.85, *p* = 0.087, *I* ^2^ = 42.05%). The fixed effects model calculated MD as −1.09 [95% CI: −1.61, −0.56]. The mean result differed significantly from zero (*z* = −4.03, *p* < 0.001). There was no publication bias according to rank correlation, Egger test, and funnel plot (*p* > 0.05) (Figure 3).

**FIGURE 4:**
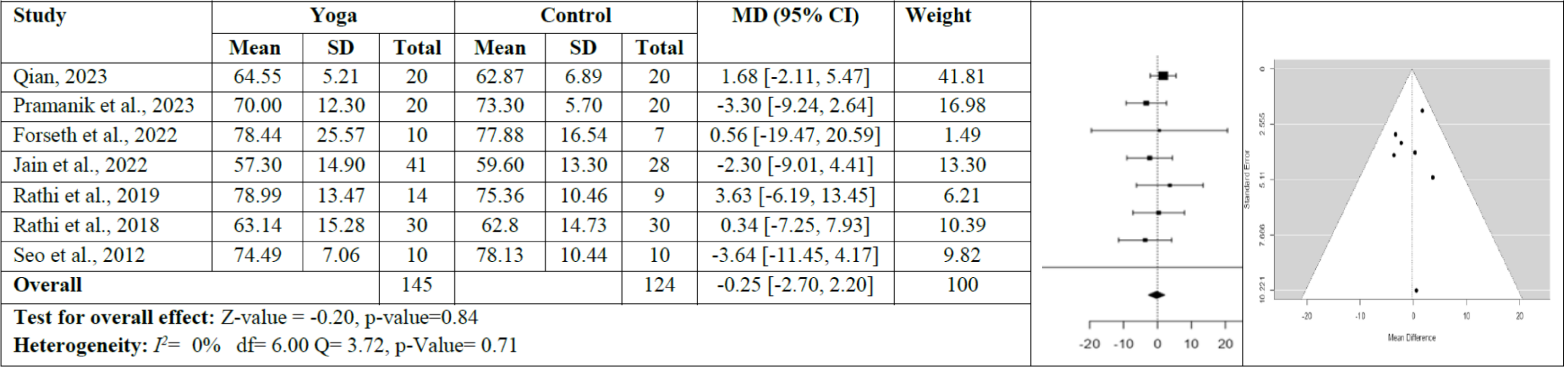
Forest plot and funnel plot for the weight of the yoga and control group.

Seven studies, including 269 participants showing the effect of yoga on weight, were included in the analysis. *Q* test and *I* ^2^ for heterogeneity was not significant (*Q*(6) = 3.72, *p* = 0.71, *I* ^2^ = 0%). The fixed effects model calculated MD as −0.25 (95% CI: −2.70, 2.20). The mean result was not significantly different from zero (*z* = −0.20, *p* = 0.84). There was no publication bias according to rank correlation, Egger test, and funnel plot (*p* > 0.05) (Figure 4).

**FIGURE 5:**
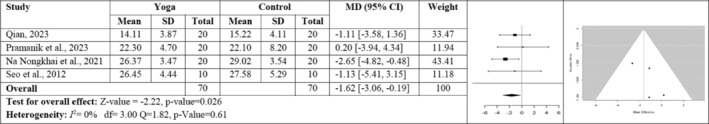
Forest plot and funnel plot for the body fat (kg) of the intervention and control group.

Four studies, including 140 participants, showing yoga's effect on body fat were analyzed. *Q* test and *I* ^2^ for heterogeneity was not significant (*Q*(3) = 1.82, *p* = 0.61, *I* ^2^ = 0%). The fixed effects model calculated MD as −1.62 (95% CI: −3.06, −0.19). The mean result differed significantly from zero (*z* = −0.22, *p* = 0.026). There was no publication bias according to rank correlation, Egger test, and funnel plot (*p* > 0.05) (Figure 5).

**FIGURE 6:**
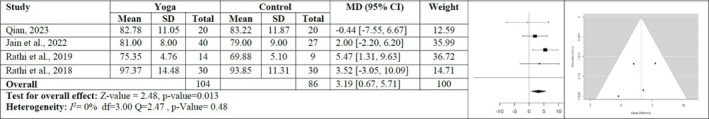
Forest plot and funnel plot for the heart rate of the intervention and control group.

Four studies, including 190 participants, showing the effect of yoga on heart rate were analyzed. *Q* test and *I* ^2^ for heterogeneity was not significant (*Q*(3) = 2.47, *p* = 0.48, *I* ^2^ = 0%). The fixed effects model calculated MD as 3.19 (95% CI: 0.67, 5.71). The mean result differed significantly from zero (*z* = 2.48, *p* = 0.01). There was no publication bias according to rank correlation, Egger test, and funnel plot (*p* > 0.05) (Figure 6).

**FIGURE 7:**
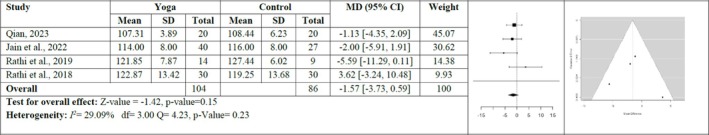
Forest plot and funnel plot for the systolic blood pressure of the intervention and control group.

Four studies, including 190 participants, showing the effect of yoga on systolic blood pressure were analyzed. *Q* test and *I* ^2^ for heterogeneity was not significant (*Q*(3) = 4.23, *p* = 0.24, *I* ^2^ = 29.09%). The fixed effects model calculated MD as −1.56 (95% CI: −3.72, 0.59). The mean result was not significantly different from zero (*z* = −1.42, *p* = 0.15). There was no publication bias according to rank correlation, Egger test, and funnel plot (*p* > 0.05) (Figure 7).

**FIGURE 8:**
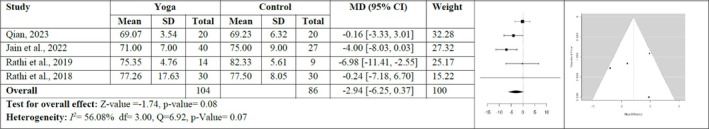
Forest plot and funnel plot for the diastolic blood pressure of the intervention and control group.

Four studies involving 190 participants showing the effect of yoga on diastolic blood pressure were analyzed. *Q* test for heterogeneity was not significant, but the *I* ^2^ value showed moderate heterogeneity (*Q*(3) = 6.92, *p* = 0.07, *I* ^2^ = 56.08%). The random effects model calculated MD as −2.94 (95% CI: −6.25, 0.37). The mean result was not significantly different from zero (*z* = −1.74, *p* = 0.08). There was no publication bias according to rank correlation, Egger test, and funnel plot (*p* > 0.05) (Figure 8).

## 4 Discussion

This systematic review and meta‐analysis identifies nine studies characterizing the effects of yoga interventions on BMI, seven on weight, four on body composition, and vital signs in children or young people with overweight/obesity. These studies compared yoga with no intervention, regular physical activity, or standard care. The findings suggest that yoga interventions positively affected BMI, body fat percentage, and heart rate, pointing to the potential benefits of incorporating yoga into lifestyle interventions for this population. The duration of yoga interventions in the included studies ranged from four to 18 weeks, indicating that even relatively short‐term programs may yield measurable benefits. However, it is essential to emphasize the need for further research to assess the long‐term sustainability and effectiveness of yoga interventions in pediatric weight management.

Evaluating study quality using the Cochrane risk of bias tool revealed considerable variability in methodological rigor across the included studies. Although seven studies reported randomization, only one provided sufficient detail regarding the randomization process, indicating a potential selection bias. Moreover, most studies raised concerns about deviations from intended interventions and outcome measurement, while a smaller proportion had incomplete outcome data. These risk of bias factors may have influenced the reliability and consistency of the reported findings and should be considered when interpreting the results.

It has been found that yoga significantly reduces BMI and body fat percentage in children or young people with overweight/obesity. This finding can be explained by yoga's effects on stress reduction, increased physical activity, and metabolic balance [34]. Similarly, consistent with the study findings, a meta‐analysis by Lauche et al. [16] also provided evidence that yoga reduces BMI in individuals with obesity. Due to the characteristics of the developmental period, young people tend to adopt a sedentary lifestyle [35]. Furthermore, it has been reported that young people have difficulty adapting to programs to increase physical activity [16]. Yoga causes less damage to the joints than exercise, so it is considered a safe method to enhance physical activity [15]. In this context, the finding that yoga decreases BMI in children and adolescents with overweight/obesity suggests that yoga could be a potential intervention for increasing physical activity in weight management programs. Although the significant results of yoga on BMI and body fat percentage (*p* < 0.05) are noteworthy, the magnitude of these effects also deserves attention. In this meta‐analysis, yoga was associated with a mean decrease of 1.09 kg/m^2^ in BMI and a 1.62% reduction in body fat percentage. While these changes may appear modest, previous research suggests that a reduction of approximately 4.8% in BMI can lead to clinically meaningful improvements in selected health outcomes in children and adolescents [36]. Compared with other physical activity interventions, the observed effects of yoga appear comparable. For example, Kelley et al. [37] found that exercise interventions resulted in a mean BMI reduction of −1.08 kg/m^2^ (3.6%) in children and adolescents with overweight or obesity. This result resembles the −1.09 kg/m^2^ effect observed in the current yoga‐focused analysis. The observed reduction in this study corresponds to a slightly smaller but potentially clinically relevant change, particularly considering the noninvasive and low‐risk nature of yoga interventions.

The effect of yoga in obesity management among adults, yoga has been reported to be more effective in reducing BMI compared with other interventions such as physical activity and calorie restriction. These studies not only highlight the contribution of physical energy expenditure through yoga but also emphasize the role of meditation in the management of obesity. [15, 38]. Yoga, which promotes health and well‐being through the integration of mind, body, and spirit, differs from conventional physical exercise by focusing not only on physical aspects but also on physiological and mental well‐being. This holistic approach is achieved through bodily postures (asanas), breathing exercises (pranayama), and meditation practices [12]. The neurophysiological mechanism underlying the effect of yoga's holistic approach on obesity management is associated with breathing regulation and the resulting balance in branches of the autonomic nervous system [39]. While intense and strenuous physical activities, as well as rapid breathing exercises, increase sympathetic activity, moderate to low‐intensity meditative practices such as various yoga postures, guided relaxation, and slow breathing reduce sympathetic arousal and enhance parasympathetic activity, thereby restoring autonomic balance. Neuropsychological mechanisms activated during this process have been found to regulate hormonal secretion from endocrine organs. Yoga interventions involving pranayama have been shown to be effective in reducing stress and stress hormones, as well as decreasing glucocorticoid secretion [40]. The studies included in the analysis did not provide a specific evaluation of the impact of mindfulness‐based guidance within yoga practices on weight management. Furthermore, it is noteworthy that there is no common standard regarding the yoga sequences implemented across these studies. This observation suggests the absence of a standardized yoga protocol that can serve as a methodological foundation in obesity management. It also highlights the need for the development of standardized protocols tailored to yoga interventions intended for obesity treatment. Without such standardization, it becomes difficult to draw robust conclusions about the efficacy of yoga in obesity management, and comparisons across studies remain limited.

As a result of the slow breathing exercises incorporated in yoga, the shift in autonomic nervous system balance from predominantly sympathetic to parasympathetic activity may lead to reductions in heart rate, as well as systolic and diastolic blood pressure [12]. In this meta‐analysis, it was found that only four studies examined the effects of yoga on heart rate and systolic and diastolic blood pressure values. In contrast with the literature, it has been found that while yoga increases heart rate, it does not affect blood pressure parameters. The inconsistency between the findings of this study and the existing literature may be attributable to variations in the yoga flow or sequencing among the studies included in the analysis. In the study, while the heterogeneity of diastolic blood pressure was high (*I* ^2^ > 50%), the heterogeneity of other outcomes was low. The high heterogeneity in diastolic blood pressure may have arisen from differences in the physiological characteristics of the participants and the influence of daily life events on blood pressure [41]. Although the overall findings suggest limited effects of yoga on blood pressure in youth, more studies with standardized measurement protocols and longer follow‐up durations are needed to assess cardiovascular outcomes more accurately.

Although the findings indicate that yoga may benefit BMI, body fat percentage, and heart rate in children and adolescents with overweight or obesity, the limited number of contributing studies and variability in methodological quality limit the generalizability of the conclusions. The current evidence is promising but insufficient to draw firm conclusions regarding the impact of yoga on weight and body composition. Therefore, there is a clear need for further high‐quality randomized controlled trials with standardized intervention protocols and rigorous reporting to better understand the potential of yoga as a supportive strategy in pediatric weight management.

### 4.1 Strengths and Limitations

One of the key strengths of this study is its focus on a specific population of children and adolescents with overweight or obesity who are often underrepresented in lifestyle intervention research. The meta‐analysis addresses an important gap in the literature by systematically analyzing studies that applied yoga interventions in this group. Furthermore, the study followed the PRISMA guidelines, applied rigorous inclusion and exclusion criteria, and employed a comprehensive search strategy across multiple databases, ensuring methodological transparency and reproducibility. However, several limitations must also be acknowledged. The relatively few eligible studies limited the statistical power and prevented subgroup analyses, such as comparisons based on intervention duration, yoga type, or age subgroups. Additionally, the limited reporting of body composition outcomes and variability in study designs may have introduced heterogeneity into the analysis. A significant limitation of this review is the high risk of bias observed in the included studies, particularly regarding randomization, deviations from intended interventions, and outcome measurement. Moreover, excluding gray literature and non‐English studies, although intended to maintain methodological quality, may have omitted potentially relevant data.

## 5 Conclusion

In conclusion, findings derived from pooled data suggest that yoga intervention is an effective method for weight management in children and adolescents with obesity. There is a need for further studies that implement yoga interventions in the literature. It is recommended that future studies examine more parameters to assess body composition. Additionally, considering the publication bias in the analyzed studies, randomized controlled trials involving yoga interventions must be based on more robust methodologies and reported in greater detail. Additionally, considering that yoga is a calming and mind–body‐based intervention, it is recommended that future studies also examine the psychological effects of yoga in children and adolescents with obesity.

## Conflicts of Interest

The authors declare no conflicts of interest.
